# Longitudinal Changes in Preschoolers’ Self-reported Psychological and Social Problems: Feasibility, Reliability, and Cross-informant Agreement

**DOI:** 10.21203/rs.3.rs-4870307/v1

**Published:** 2024-09-02

**Authors:** Meingold Hiu-ming Chan, Xin Feng, Yihui Gong, Karis Inboden

**Affiliations:** University of British Columbia; The Ohio State University; The Ohio State University; The Ohio State University

**Keywords:** Berkeley puppet interview, psychological problems, social problems, reliability, cross-informant agreement, child report

## Abstract

For decades, parental report was used to assess children’s psychological symptoms and social problems. The Berkeley Puppet Interview (BPI) utilizes hand puppets to collect questionnaire-style data from children, allowing consideration of children’s own perspective. The current longitudinal study compared the feasibility and reliability of preschoolers’ self-report with BPI at age 4 (*M* = 4.03, *SD* =0.16; 52% boy, 82% White American) and 5 (*M* = 5.22, *SD* = 0.36; 51% boy, 85% White American) as well as cross-informant agreement among children, mothers (74% above college education), alternate caregivers (>90% biological fathers), and coders. Children completed symptomatology, social, and parenting scales of BPI and their parents completed surveys assessing similar constructs. Our findings revealed both similarities and changes across ages. Specifically, the reliability and cross-informant agreement of the broad symptomatology and parenting scales were promising at both timepoints; however, 4-year-olds showed lower internal consistency in social scales.

## Introduction

### Longitudinal Changes in Preschoolers’ Self-reported Psychological and Social Problems:

#### Feasibility, Reliability, and Cross-informant Agreement

Children’s socioemotional adjustment at a young age is an important predictor to a wide range of life outcomes including mental and physical health, academic achievements, and interpersonal relationships (Denham et al., 2015; Nigg, 2017). Hence, early identification of socioemotional problems such as internalizing and externalizing behavioral problems as well as social competence in preschool children is critical for timely interventions. For decades, developmental research has relied on parental reports, especially maternal, to understand children’s socioemotional development and parenting due to the lack of suitable tools to assess children’s perspective at preschool age. The Berkeley Puppet Interview (BPI) is a valuable and innovative tool that uses hand puppets to obtain questionnaire type data from young children regarding their perceptions of their own socioemotional functioning (Measelle et al., 1998). Recent studies have adopted this age-appropriate method and gained important insight into child development (Luby et al., 2007). A vast body of studies have shown adequate internal consistency and validity of BPI scales among children around 5 years old (Coldwell et al., 2006; Measelle et al., 1998; Ringoot et al., 2013; Stone et al., 2014). However, very few studies have used BPI with children younger than 5 years old and it is unclear whether or how the performance of children changed between ages 4 and 5. Further, limited studies have used both mother- and father-reports to evaluate the cross-informant agreement of child-reported psychological and social problems using BPI scales, especially the parenting scale. Hence, the current study examined the changes in feasibility, reliability, and cross-informant agreement of child-reported psychological symptoms and social problems between ages 4 and 5 using multi-informant data from mothers, alternate caregivers (AC), and coders.

### The Importance of Preschoolers as Informants & Cross-informant Perspectives

The majority of past developmental research on young children has relied heavily on maternal reports using questionnaires while perspectives from other informants in the family such as fathers were often overlooked (Martel et al., 2017). Informed by the family system perspective, multi-informant data collection including multiple family members has been encouraged in recent years to obtain a more comprehensive understanding of child development and parenting (Renk, 2005; Sessa et al., 2001). Interestingly, correlations between reports from multiple informants such as father, mother, and teacher are generally low to moderate, reflecting different perspectives and contexts (De Los Reyes et al., 2015).

Despite the increasing likelihood of collecting multi-informant data, data from young children are still rarely obtained due to concerns about whether young children could reliably report their own behaviors and experiences given potential challenges, such as short attention spans, low verbal abilities, and response biases (Measelle et al., 1998; Ringoot et al., 2013). Although structured verbal interviews were developed to aid children to share their experience, especially in the context when children’s testimony is essential (Hershkowitz et al., 2012), some open-ended interviews requiring higher level of verbal ability and long attention span may not always be the most suitable method (Miller & Aloise, 1989). Therefore, researchers have been exploring other methods that are more appropriate for children. Methods like interviews using puppets or storytelling using toy props have been proposed. However, their appropriateness for gaining reliable and valid reports from children is still an active research area. Increasing empirical research has found that children could be reliable reporters of their experiences when puppets are utilized, demonstrating the possibility of obtaining self-report from children (Bretherton & Oppenheim, 2003; Measelle et al., 1998; Sessa et al., 2001).

Assessing children’s self-perception of their own behaviors and family environment is key. First, studies have suggested that children’s internalizing behaviors may be more difficult for parents or teachers to detect and more likely to be identified by children themselves (Stone et al., 2014). Thus, it may be critical to obtain self-report of children’s internalizing problems for early identification. Second, past research has shown that children’s perception of parenting and family relationships are important predictors of their later development (Ablow et al., 2009). Therefore, it is crucial to assess children’s perception in addition to parent’s own subjective perception of their parenting behaviors or marital relationship. Interestingly, Sessa et al. (2001) indicated that the child-report and observer’s rating of mother’s parenting were more closely related than that between child- and mother-report as well as observer’s rating and mother-report. As such, there might be potential biases in mothers’ self-reporting on their own parenting, supporting the importance of child-report and cross-informant perspectives on parenting.

The BPI was designed to assess self-perception of own emotional and behavioral problems, social and school functioning, as well as family environment among young children between age 4 and 8 (Albow et al., 1999; Ringoot et al., 2013). The design of BPI aimed at overcoming challenges in collecting reliable data from young children. Specifically, given that children generally favor toys over paper and pencil measures, the BPI utilizes puppets to maintain attention in children (Greenspan, 2008; Measelle et al., 1998). Past studies have found that puppets could keep young children engaged in the interview by creating a dynamic interaction between the child and the interviewers (Eder, 1989). Additionally, the puppets in the interview act as peers of children hence minimizing the sense of judgement from adults and create a comfortable environment for children to provide honest responses. Interviewers were also instructed to use vocabulary and language that matches with the language ability of young children. In addition to this age-appropriate design, scales of BPI were also carefully developed. The original paper on BPI published in 1998 provided a detailed account of how the items and scales in BPI were constructed (Measelle et al., 1998). Principal component analyses (Albow et al., 1999; Measelle et al., 1998; Sessa et al., 2001) as well as confirmatory factor analyses (Jia et al., 2016; Ringoot et al., 2013) were conducted to support the development of a number of scales, including symptomatology, social, and academic scales. The BPI has been used in over hundreds of peer-reviewed studies across multiple countries and translated to seven different languages. The vast body of literature using the BPI has collectively shown that the BPI is a feasible tool to obtain self-report from children at age 5 to 8. Nonetheless, the feasibility and reliability of BPI at an even younger age as well as longitudinal changes in child-reporting are underexplored.

### Developmental Perspectives on Preschoolers’ Self-reporting

The evaluation of whether the BPI is an age-appropriate tool for younger preschoolers warrants a developmental perspective since children’s ability to self-report their emotions, behaviors, and social surroundings depends largely on their cognitive development. Given the rapid development in the first few years of life, even with a one-year age difference, there could be major developmental changes that may lead to potential differences in the ability of self-reporting accurately and reliably. Here we review developmental changes between the fourth and fifth birthdays that could be relevant to their performance in BPI, which include three broad aspects – social cognition, executive function, and language development.

Important developmental milestones in social cognition around the age of four to five include the development of the theory of mind, the understanding of self and others’ mental states. At around age 4.5 children typically start to develop a false belief understanding that one can hold a belief that is different from the reality (Wellman & Liu, 2004). The understanding of own and others’ mental states is essential for self-reporting in the BPI since it requires children to be introspective of own thoughts and feelings as well as being aware of others’ thoughts and feelings in daily life. Relatedly, preschoolers also develop their emotion understanding, which includes the ability to identify, predict, and explain their own and others’ emotions (Harris, 2008). Although children at age 4 demonstrate some emotion understanding, children older than four may be better able to understand intrapersonal and interpersonal factors underlying their own emotional experience, which may facilitate children’s self-understanding and ability to accurately report their own emotions and behaviors.

Developmental changes in executive functioning, including working memory and inhibitory control, could also result in differences in BPI performance across four and five-year-olds. According to a comprehensive review on executive function (Best & Miller, 2010), a linear improvement in working memory task can be observed from ages 4 to 14. Therefore, it is possible that 5-year-olds can hold longer statements in memory than 4-year-olds and hence perform better in BPI, especially for longer items. Additionally, although most children can learn what responses are acceptable from the practice items and avoid using non-codable responses (e.g., just “yes/no”) for subsequent items, children who have poor inhibitory control tend to show difficulties in inhibiting their prepotent and often still respond with non-codable response. Inhibitory control can also contribute to children’s concentration and attention during the BPI. Thus, it is important to investigate the feasibility of using the BPI with younger children whose inhibitory control and concentration are still undergoing rapid development. Lastly, at the age of four, most children have already developed the ability to use pronouns, take turns in conversation, as well as understand questions, negative sentences, and compound sentences (Tyack & Ingram, 1977; Visser-Bochane et al., 2020), which are all essential for completing the BPI. However, between ages 4 and 5, children also begin to be able to produce compound sentences themselves and become more intelligible in their speech (Visser-Bochane et al., 2020).

Together, important developments in social cognition, executive function, and language/comprehension between children’s fourth and fifth birthdays play important roles in determining their ability to self-report through BPI. Conceivably, due to developmental differences, children may perform less optimally at age 4 than 5 but it is unclear whether 4-year-olds still pose the sufficient basic ability for completing the BPI and producing reliable responses.

### Limited Studies on Reliability and Cross-informant Agreement of Preschoolers’ Self-report

Most past research adopting the BPI primarily utilized the symptomatology scales, which assessed children’s internalizing symptoms including depression, separation anxiety, over-anxiousness, and externalizing symptoms such as oppositionality and defiance, overt aggression/hostility, conduct problems, and relational aggression. The psychometric properties of the symptomatology scales among children aged 5 to 8 have been examined. Research showed acceptable internal consistency for the overall symptomatology scale (Howland et al., 2016; Huber et al., 2019; Measelle et al., 1998) but reported mixed results for internal consistency for individual subscales (e.g., Cronbach’s alphas ranged from 0.36 to 0.81) (Albow et al., 1999; Arseneault et al., 2005). Although some symptomatology subscales have been reported with low internal consistency in certain study (Albow et al., 1999), the BPI is still one of the very few tools that can assess common psychological symptoms, including depression, anxiety, and aggression, in young children. Hence, it is valuable to examine the reliability of these scales in different samples to provide more empirical findings to guide informed decision on the use of the scale in future studies. The test-retest reliability and inter-rater reliability on the symptomatology scales are good (0.71 and 0.90, respectively) (Arseneault et al., 2005). Past studies have also found significant and positive correlation between mothers and child’s report on children’s internalizing and externalizing problems (Luby et al., 2007; Ringoot et al., 2013, 2017). Yet, limited studies have examined the agreement between child-report and reports from caregivers other than mothers as well as the longitudinal stability, if any, of child-report via BPI.

Aside from the symptomatology scales, BPI also includes social scales to assess children’s own perception of social competence and peer acceptance. A total of seven scales are included under social (or socioemotional) scales, including prosocial, peer acceptance/rejection, social inhibition/extraversion, bullied by peers, asocial with peers, emotion regulation, and emotion knowledge (Ablow & Measelle, 2018), among which prosocial behavior and social acceptance were included in the current study. Limited research has tested the reliability and validity of these new social scales in BPI. One study has tested the reliability of these new social scales with children at ages 5 to 7 and reported acceptable Cronbach’s alphas of peer acceptance (.68-.79) (Ringoot et al., 2013). Another study has shown a good reliability (Cronbach’s alphas = .70-.73) for the prosocial behavior subscale with children of 4 years 5 months to 5 years 3 months (Huber et al., 2019). However, the validity or cross-informant agreement of the social scales is underexplored. The BPI parenting scale is also developed recently to assess children’s perception of their parents’ behaviors (Ablow & Measelle, 2018). The scale included a total of six subscales. The current study focused on the warmth/enjoyment and anger/hostility subscales since some studies have already reported an acceptable reliability with young children (Coldwell et al., 2006; Sessa et al., 2001) and found agreement with parental reports of anger (Coldwell et al., 2006; Pike et al., 2016). However, it is unclear whether child-report parenting behaviors in BPI are associated with mothers’ and fathers’ own reporting of parenting.

Overall, there are limited reports on the feasibility, reliability, and cross-informant agreements of the BPI scales when used with younger preschoolers. Thus far, only four papers have reported the use of the BPI among children younger than 5-year-old (Kuntsche, 2017; Luby et al., 2002, 2007; Sessa et al., 2001). Luby et al. (2002, 2007) and Sessa et al. (2001) used the BPI symptomatology and social scales, respectively, while Kuntsche (2017) designed a new scale to assess children’s perception of adult’s alcohol use and mood. Although some of these papers suggested reasonable reliability (Cronbach’s alpha > .70; Sessa et al., 2001) and validity (Luby et al., 2002) when using the BPI with children as young as four, which is a promising start, more thorough investigation of the similarities and differences in BPI performance between children of age 4 and 5 are needed to inform future use of BPI with younger children.

#### The Current Study

Ample research has indicated that BPI could generate reliable and valid data from children above 5 years of age, yet there are very few studies that explore the potential of using BPI in children younger than age 5. It is unclear how the performance of children in BPI may differ between the age of 4 and 5 given their developmental changes. Although the performance of younger children’s self-reporting may be less optimal, it would still be informative to identify scales that may be appropriate to use with younger children given the importance of early identification of psychological symptoms and obtaining children’s perspective on their experience to providing suitable intervention. The current study examined the feasibility, reliability, and cross-informant agreements of the symptomatology, social, and parenting scales of BPI among young preschool children at two time points (mean age was 4.03 at time 1 and 5.22 at time 2). To evaluate feasibility, we reported the percentage of completion and codable responses. We also examined the global impression ratings on the level of believability, consistency, social desirability, and concentration of children’s responses as well as their level of distress during BPI. Two types of reliability were evaluated: internal consistency across items within each subscale and inter-rater reliability. Stability between responses at age 4 and age 5 was also assessed though mostly exploratory. Evidence of stability in children’s internalizing/externalizing symptoms have been found in past studies but not always consistent (Basten et al., 2016). Furthermore, it is unclear whether children’s self-report of social and parental environment is stable over time. Cross-informant agreement was examined based on the association between the child-report on BPI scales and both mother and AC’s ratings on similar constructs. We compared the feasibility, reliability, and cross-informant agreements of child-report across the two timepoints to gain insight into the changes between ages 4 and 5.

About half of the children in our sample had mothers who met the clinical criteria of major depressive disorder (MDD). Considering that depressed mothers’ report on their children’s behaviors tend to be negatively biased (Ordway, 2011), acquiring the perception from the child and the AC could be particularly valuable. Although we acknowledge that children with depressed mothers may have a higher risk of behavioral and emotional problems, the focus of the current study was to evaluate the performance of BPI at different ages, so we reframed from comparing the differences in BPI scores between the depressed and nondepressed groups before the appropriateness of this tool has been established.

## Method

### Participants

Data for the current study were drawn from a larger longitudinal study that investigated the association between young children’s socioemotional development and maternal depression. This study was approved by the Institutional review Board (IRB) of the institution. This study was not preregistered. Mothers were recruited through online advertisement as well as through flyers sent to preschools, daycare centers, and clinics from a large Midwest city. Mothers were eligible if they (1) were 21 years or older; (2) had a biological child between 3.5 and 4 years; and (3) did not meet the clinical criteria of psychosis, manic disorders, and substance abuse in the past 6 months assessed using the Structured Clinical Interview for DSM-5 Research Version (SCID-5-RV) (First, 2014). Mothers were assigned to the depressed group if they met the criteria of MDD during the child’s lifetime in SCID-5-RV in screening, and those who did not have MDD during the child’s lifetime were assigned to the control group. The group assignment was kept the same for T1 and T2. Children were excluded if they had been diagnosed with a developmental delay/disorder or had an IQ score below 70 in the WPPSI^™^-IV Wechsler Preschool & Primary Scale of Intelligence^™^ | Fourth Edition (WPPSI-IV) in screening. Once both mothers and children were determined as eligible for the study, mothers were asked to nominate an AC. The AC was the mothers’ partner if she is married or cohabiting, or a grandparent or other relative who knows the child well if the mother was not married or cohabiting (91% and 93% were biological fathers at T1 and T2, respectively).

At time 1 (T1), 125 mother-child dyads completed the visit, however two dyads were excluded from analyses due to missing impression code and completing T1 visit after age 4.5, which is 3 standard deviations older than the mean age, respectively. Therefore, a final sample of 123 mother-child dyads (52.0% boy; *M*_age_ = 4.03, *SD* = 0.16, range = 3.49–4.47) were included in the analyses. At time 2 (T2), 91 of the 123 dyads returned for the lab visit. Among which, video recording system failed for two of the visits and hence no BPI coding could be done. Thus, a final sample of 89 mother-child dyads (51.0% boy; *M*_age_ = 5.22, *SD* = 0.36, range = 4.64–6.37) were included in the analyses. A 73% retention rate is within the typical range reported in longitudinal study (Teague et al., 2018), especially considering T2 data collection was completed during the pandemic. Little’s MCAR test showed that the data were missing completely at random, *c*^2^ (35463) = 5704.43, *p*>.05. Full sample characteristics at T1 and T2 were presented in Supplementary Table S1.

#### Procedure & Measures

Children were screened for cognitive ability, including verbal and spatial ability, using WPPSI-IV to ensure that they could complete some of the more cognitively demanding tasks in the visit. Eligible mother-child dyads completed a 2.5- to 3-hour lab visit (with the exception of 11 families who completed the T2 visit online via Zoom due to the pandemic), including BPI. Mothers and ACs also completed an online survey prior to the lab visit.

### The Berkeley Puppet Interview

The BPI was an instrument designed to obtain self-reports of young children’s own psychological development and social experience under 7 domains: symptom, social/emotional, academic/cognitive, personality, parenting, sibling, and marital relationship scale (Ablow & Measelle, 2018; Measelle et al., 1998; Measelle & Ablow, 2019). Researchers could select scales that fit with their research questions. All three authors and two undergraduate research assistants (RA) received a 2-day training workshop from Dr. Measelle on how to administer and code the BPI. The first and third authors received additional training to be trainers and trained other undergraduate RAs after being certified to train others. Throughout the process of research design and data collection, the authors have maintained communication with Dr. Measelle and his team on choosing scales based on their relevance to the study aims and whether the scale was potentially suitable for children younger than age 4.5 as well as addressing issues that come up during data collection or coding. After careful consideration, ten scales were selected for the current study. These included depression (7 items), separation anxiety (6 items), overanxious (7 items), oppositional defiant problem (ODP) (6 items), overt hostility/aggression symptoms (7 items), prosocial behaviors (7 items), peer acceptance/rejection (5 items), social inhibition (6 items), and parental emotional warmth (6 items for mother, 6 items for fathers) and hostility (6 items for mother, 6 items for fathers). Four broad scales were constructed at both time points: internalizing (including depression, separation anxiety, and overanxious), externalizing (ODP and aggression), social competence (prosocial behaviors, peer acceptance/rejection, and social inhibition), and parenting (emotional warmth and hostility). The selection of the ten scales resulted in a 75-item interview in the study. At T2, the same set of items were administered, except the ODP and peer acceptance/rejection scales and two items from the parental hostility scale (item 2 and 26) were taken out for the online study (*n* = 11) due to the difficulty of maintaining children’s concentration over video call. These decisions were made after inspecting the internal reliability of T1. The ODP and peer acceptance/rejection scales both had Cronbach’s alpha below .60 at T1 and could not be improved by removing any item(s). When we examined the inter-item correlation matrices and the item-to-scale correlations in the parental hostility scale, items #2 and #26 had low correlations with other items and the scale, and the removal of these items improved the scale reliability substantially, hence these items were removed.

Past studies reported adequate Cronbach’s alphas for all symptomatology scales, except that the depression subscale reported in some studies was relatively low (e.g., Cronbach’s alpha = .36 and .44 in Albow et al. (1999) and Ringoot et al. (2013), respectively). The social scales also showed acceptable reliability of Cronbach’s alpha ranged from .65 to .70 in difference studies across ages 4.6 to 7 (Huber et al., 2019; Measelle et al., 1998; Ringoot et al., 2013). Lastly, some recent studies using the parenting scales revealed acceptable reliability (alphas = .68 to .77) (Sessa et al., 2001) whereas some reported poorer reliability (alphas = .56 to .66) (Coldwell et al., 2006); both with a mean age of 5.

#### Procedure.

The child was first introduced to Iggy and Ziggy, the two identical dog hand puppets used for BPI, as well as the basic format of the interview. The puppets were positioned at the child’s eye level obscuring the interviewer’s face so the child could focus on interacting with the puppets rather than the interviewer. Throughout the interview, the two puppets presented a series of opposing statements (e.g., “I cry a lot” and “I don’t cry a lot”) and subsequently invited the child to tell them which statement best describes them (“how about you?”). Interviewers were trained to use child-appropriate language when they engaged with the child using the puppets. In this way, children could engage with the puppets in a relatively natural conversation with their “peers”. Interviewers were also instructed to use their natural voice and use neutral and warm tone when presenting both positive and negative statements to avoid influencing children’s responses. Children can respond in ways that are comfortable to them, but interviewers would prompt in a standardized way if the response was ambiguous or unclear. The positive and negative statements were counterbalanced so that half of the items were led with positive statement and the other half with negative statement. To reduce fatigue, the interviews were broken down into two sections and were separated by games and activities.

#### Coding.

The interviews were video recorded and coded later. Based on an existing coding manual (Ablow & Measelle, 2018), all responses were coded on a 7-point scale, with lower score indicating more negative responses. The code assigned to the child’s response depended on the degree to which the child endorsed the positive or negative statement presented to them. Responses were deemed not codable if the child gave an unclear or inaudible response or the child clearly misunderstood the questions and gave an irrelevant response. Coders also rated children’s overall performance on the level of believability, consistency, social desirability, concentration, and distress children exhibited during BPI with one-item for each dimension on a 7-point scale, with lower score indicated poorer performance. This tool was recommended to the research team by Dr. Measelle as another way to examine the quality of the data collected by BPI. Full details of coding and global impression coding can be found in Supplementary Material S4 and S5. The first author trained a team of graduate and undergraduate research assistants and provided master coding files for the research assistants to test coding reliability. The coding team met weekly to discuss any coding questions or disagreements. Twenty percent of the interviews were double coded for inter-rater reliability.

### Child Behavioral Checklist

Parental reports on children’s emotional and behavioral problems were assessed using the Child Behavior Checklist for Ages 3 to 7 (Achenbach et al., 2001), a 99-item questionnaire on a 3-point rating scale. The questionnaire includes a total of seven subscales as well as six DSM-oriented subscales. Specifically, the sum scores of anxious/depressed (8 items), aggressive behaviors (19 items), DSM-oriented affective problems (10 items), DSM-oriented anxiety problems (10 items), and DSM-oriented ODP (6 items) subscales were used in the current study since these subscales measured similar construct to the child-report depression, anxiety, and aggression symptoms in BPI. The internal reliability of these subscales was all acceptable. At T1, Cronbach’s alphas for these subscales were .65, .89, .61, .70, and .87, respectively for mothers and .66, .89, .64, .70, and .82, respectively for ACs. At T2, Cronbach’s alphas were .73, .90, .60, .77, and .83, respectively for mothers and .74, .89, .71, .66, and .83, respectively for ACs.

### Children’s Behavioral Questionnaire

The Children’s Behavioral Questionnaire (CBQ) (Rothbart et al., 2001) assessed parental report on children’s temperamental disposition, with 94 questions rating on a 7-point scale (1 = *not at all frequently,* 7 = *very frequently*). The sum scores of the subscales of anger/frustration (6 items), sadness (7 items), and shyness (6 items), which measured similar constructs as the BPI symptom and social scales, were used in the current study. The internal reliabilities of these subscales were acceptable. At T1, Cronbach’s alphas for these subscales were .79, .69, and .87, respectively for mothers and .78, .63, and .84 for ACs. At T2, Cronbach’s alphas were .73, .62, and .84, respectively for mothers and .75, .56, and .87 for ACs.

### Parenting Behaviors and Dimensions Questionnaire

Parental reports of their own parenting behaviors were assessed using the Parenting Behaviors and Dimensions Questionnaire (PBDQ) (Reid et al., 2015), with 33 items on a 6-point rating scale (1 = *never,* 6 = *always*). The mean scores of the emotional warmth (6 items), punitive discipline (5 items), permissive (7 items), and democratic discipline (5 items) subscales were used in the current study as they measure similar construct as the child report parenting behaviors in BPI. At T1, Cronbach’s alphas for these subscales were .69, .71, .59, and .71, respectively for mothers and .76, .76, .69, and .74 for ACs. At T2, Cronbach’s alphas were .81, .67, .54, and .80, respectively for mothers and .74, .77, .76, and .65 for ACs.

### Demographic information

Mothers reported children’s age and sex as well as their own race, family income, and educational level, while ACs reported their relationship with the child in the online survey.

### Data analysis

Feasibility was evaluated by the percentage of children who completed the interview (less than 25% of items were skipped) and the percentage of not codable responses on item level. We also assessed the differences in mean scores of global impression code on the level of believability, consistency, social desirability, concentration, and distress displayed by children during BPI across timepoints using paired sample t-test. The inter-rater reliability of these scores were assessed with intra-class correlation (ICC). Internal consistency reliability was reported using Cronbach’s alpha. Inter-rater reliability was reported with ICC. Stability over time was assessed using Pearson’s bivariate correlation between the scores of the same BPI scales at T1 and T2. To test cross-informant agreement, correlations between children’s self-report on four broad scales and ten subscales in BPI and parental report on CBCL, CBQ, and PBDQ were examined using Pearson’s correlation. The BPI scales under symptomatology were reversed coded for these analyses (i.e., higher score indicates more problems) for easier interpretation. Study material and analysis codes will be provided upon reasonable request. Data for this study are not available for sharing because data coding has not been completed.

## Results

### Feasibility & Descriptive Statistics

The feasibility of the BPI was evaluated by the amount of codable data and children’s performance rated by coders during the interview. At T1, all children were willing to do the interview. Of which, 80% of the children finished the whole interview, while the remaining 20% had more than 25% of the items skipped due to time constraints or children losing focus to the interview. Across items, an average of 7.02% (*SD* = 4.72%) of the children’s responses were determined to be not codable. The percentage of not codable responses ranged from 1.60% (items 13, 16, 53, and 58) to 29.60% (item 18: My mom says she loves me) for individual items. The high percentage of not codable responses in item 18 were due to large number of children responding “My mom loves me” rather than whether or not their mother *says* she loves them, which the authors have decided that this response was not clear enough to code for mothers’ emotional warmth. Global impression ratings of children’s performance by the coders indicated that children showed moderate level of believability, concentration, understanding, and consistency as well as low level of anxiety and distress (see Table 1). The inter-rater reliabilities for the global impression ratings were within acceptable range (ICCs = .52 to .91 at T1 and .78 to 1 at T2).

At T2, all children were willing to do the BPI task and only one child was not able to complete the whole interview. Across items, an average of 1.24% (*SD* = 1.10%) of children’s responses were not codable, ranging from 0% (40 out of 81 items) to 3.33% (Q17, 31, 36, 38, 67). When comparing children’s performance between T2 and T1, paired sample t-test showed that the global impression ratings of children’s level of believability, concentration, understanding, and consistency were statistically significantly higher at T2 than T1 (see Table 1). The mean and standard deviation for the four broad scales and each individual subscale were presented in Table 2.

### Reliability

Internal consistencies of all scales were measured with Cronbach’s alpha and presented in Table 1. At both timepoints, all broad scales showed good reliability (Cronbach’s alphas > .70) except social scales at T1. Overall, the internal consistencies of most subscales improved from T1 to T2, except overt hostility/aggression and maternal hostility. At T1, most subscales had low reliability; only overt hostility/aggression had Cronbach’s alphas above .70, while prosocial behaviors, father emotional warmth, and mother hostility had a Cronbach’s alpha at or above .60. At T2, two individual subscales had Cronbach’s alphas above .70, including prosocial behavior and mother emotional warmth and several had Cronbach’s alpha at or above .60, such as separation anxiety, social inhibition, and peer acceptance. Inter-coder reliabilities assessed by ICC were above .90 for all scales at both T1 and T2 (full details are in supplementary Table S2).

### Stability Over Time

Pearson’s correlations between the same broad scales and subscales across T1 and T2 were presented in Table 1. Correlations of .1 were considered weak, .3 moderate, and .5 or higher strong (Akoglu, 2018). Social and parenting broad scales exhibited significant moderate correlations over time (*rs* = .34 and .35, respectively). Among individual scales, significant weak to moderate correlations over time were only found for separation anxiety, prosocial behaviors, father emotional warmth, and mother hostility scales (*rs* ranged from .23 to .41).

### Cross-informant Agreement

Cross-informant agreement was evaluated based on Pearson’s correlations between BPI scales and children’s related emotional, behavioral, social problems as well as parenting behaviors rated by mothers and ACs. The full correlation matrix was presented in Supplementary Table S3. In the following, only correlations between related constructs were summarized.

At T1, more positive child-reports of general parenting experience were significantly correlated with maternal reports of lower levels of aggression (*r* = − .24, *p* = .008) and DSM-oriented ODP (*r* = − .25, *p* = .006). Similar associations were observed with child-reported AC emotional warmth and maternal reported aggression and ODP (*r* = − .27, *p* = .003 and *r* = − .22, *p* = .015, respectively). Among the five subscales under symptomatology, BPI separation anxiety scale was positively correlated with mother-reported anxious/depressed symptoms (*r* = .20, *p* = .030) and DSM-oriented affective problems (*r* = .21, *p* = .020). Among the three social subscales, higher score (fewer problems) of BPI peer acceptance/rejection was significantly correlated with fewer mother-reported aggressive behaviors (*r* = −.19, *p* = .038) and DSM-oriented ODP (*r* = − .20, *p* = .030). However, none of the broad scales on BPI were significantly correlated with directly related constructs reported by mothers.

In terms of BPI association with ACs’ reports at T1, higher level of child-reported broad internalizing symptoms scale and separation anxiety were significantly linked to AC-report of higher level of temperamental sadness (*r* = 0.21, *p* = .028 and *r* = .23, *p* = .017, respectively). BPI parenting broad scale was significantly correlated with higher level of emotional warmth reported by ACs (*r* = .20, p=.037). Relatedly, for parenting subscales, child report of father emotional warmth was significantly and positively correlated with AC’s reported emotional warmth (*r* = .20, *p* = .044) and democratic discipline (*r* = .24, *p* = .012). Interestingly, children’s report of father hostility was negatively correlated with AC’s report of punitive discipline (*r* = −.19, *p* = .047). Furthermore, child report of mother’s parenting was also linked to AC report of parenting. Specifically, higher level of child-reported mother emotional warmth is correlated with higher level of AC-reported democratic discipline (*r* = .23, *p* = .015), while child-reported maternal hostility is positively linked to AC-reported permissive parenting (*r* = .025, *p* = .011).

At T2, there were some scales that demonstrated agreement among all three informants. Interestingly, the BPI depressive scale was positively associated with both mothers’ and ACs’ rating on aggressive behaviors (*r* = .35, *p* = .001 and *r* = .26, *p* = .020, respectively), DSM-oriented ODP (*r* = .36, *p* = .001 and *r* = .40, *p*<.001, respectively), and temperamental anger (*r* = .26, *p* = .016 and *r* = .25, *p* = .022, respectively). For social subscales, we found that higher level of social inhibition (lower score) in BPI was significantly related to more DSM-oriented anxiety problem reported by both mothers and ACs (*r* = * −* .23, *p* = .033 and *r* = *−* .24, *p* = .031).

Some BPI scales were only significantly associated with mother-report at T2, but not with AC report. For examples, mother-report of DSM-oriented affective problems were significantly related to more symptoms reported by children in BPI internalizing broad scale (*r* = .34, *p* = .001), and four symptomatology subscales, including depressive scale, separation anxiety, overanxious, and ODP (*r* = .24 – .29, *p* = .006 – .025), as well as more problems (lower score) in social inhibition (*r* = *−* .30, *p* = .005). Child-reported ODP were also significantly and positively correlated with maternal reports of aggressive behaviors (*r* = .22, *p* = .046).

Interestingly, more BPI scales were significantly correlated with reports by ACs only, but not with maternal report, at T2. For instance, higher level of BPI externalizing broad scale was associated with more AC-reported symptoms on DSM-oriented ODP (*r* = .36, *p* = .001). Higher level of separation anxiety reported by children in BPI was significantly related to higher level of AC reported temperamental shyness (*r* = .26, *p* = .020). Likewise, higher levels of aggression and ODP reported by children were also significantly correlated with higher level of DSM-oriented ODP reported by ACs (*r* = .23, *p* = .039 and *r* = .32, *p* = .005, respectively); similarly, more child-reported problems (lower score) in peer acceptance/rejection were related to higher AC-reported ODP and aggression (*r* = − .27, *p* = .020 and *r* = −.30, *p* = .008, respectively). Furthermore, lower score (more problems) in BPI social scale was significantly correlated with more aggressive behaviors (*r* = − .23, *p* = .037) and temperamental anger (*r* = −.28, *p* = .011). More problems (lower scores) of child-reported social inhibition were linked to higher levels of AC reported DSM-oriented anxiety symptoms (*r* = − .24, *p* = .029) as well as temperamental shyness (*r* = − .27, *p* = .015). For parenting scales, BPI parenting broad scale was positively related to AC-reported permissive parenting (*r* = .37, *p* = .004). We also found a significant positive correlation between child-reported father emotional warmth and hostility with AC-reported permissive parenting (*r* = .29, *p* = .030 and *r* = .46, p < .001, respectively).

## Discussion

The current study is one of the first studies that compare the BPI performances at ages 4 and 5 in the same group of children to provide insight into how children’s performance in BPI could change longitudinally. Furthermore, our multi-informant data allowed us to shed light on cross-informant agreement. Our study revealed both similarities and differences in BPI performance as children aged from age four to five. Children at both ages gave internally consistent responses within the internalizing, externalizing, and parenting broad scales; the social and parenting broad scales also showed stability over time. Good cross-informant agreement at both ages were found for the parenting broad scale. We discussed the differences in BPI performance between the two ages considering important developmental changes.

### Similarities and Differences in BPI Performances Between Ages 4 and 5

#### Feasibility

The current study showed that there was a high percentage of children completing the whole interview and low percentage of not codable responses in BPI not only when children were at 5 years old, but also when they were at the age of 4. Coders also rated children at T1 with a moderate level of believability and understanding of the items and that children only exhibited low levels of distress. Additionally, it is encouraging to see that all BPI scales demonstrated good inter-rater reliability at both timepoints, suggesting that the training and existing coding systems are applicable to responses from children at both ages. As such, it is feasible to use the BPI even at the age of 4, which is in line with past studies that used BPI with children younger than 5 years old (Kuntsche, 2017; Luby et al., 2002, 2007; Sessa et al., 2001). Unsurprisingly, the completion rate and percentage of codable responses at age 4 were lower than that at 5. Children at age 4 who could not complete the whole interview were generally distracted by other objects in the room and hence could not focus on the interview. At times, they demonstrated strong incompliance that led to ending the interview earlier. These behaviors could be explained by the lower inhibitory control and shorter attention span observed in younger children, supported by the global impression codes.

The higher non-codable responses at age 4 than one year later can be attributed to children’s poorer ability in understanding more complex, compound sentences (Visser-Bochane et al., 2020). For examples, several items under the social scales using the structure of “When/If…, I …”, which may be hard for children to understand or remember. Researchers who are interested in using the BPI with younger children may consider avoiding these items that may not be developmentally appropriate for 4-year-olds given their language development.

#### Reliability

At both timepoints, all scales had good inter-coder reliabilities and broad scales of internalizing symptoms, externalizing symptoms, and parenting scales revealed good internal consistency. This suggested that children at both ages demonstrated the ability to provide consistent reports on their symptomatology and perception of parenting. However, Cronbach’s alpha of the broad social scale was considerably low at T1. A possible explanation behind this low internal consistency is that the theory of mind of children at age four is still undergoing development (Wellman & Liu, 2004). Some of the items in social scales may require children’s ability to distinguish own mental states and others (e.g., “I worry that other kids don’t like me”) as well as ability to speculate others’ mental states (e.g., “other kids like to play with me”). Although some of the items in the parenting scales may also have similar demands, children at this age typically spend more time with their parents than peers, thus it may be easier for children to draw onto their experience with their parents as compared to interactions with peers. In fact, interviewers in our study sometimes had to help children think of contexts that they meet other kids (e.g., when they go to the playground) since some of them did not attend preschool or kindergarten. The limited social interactions could also potentially impact the children’s ability to report their self-perception of social competence consistently. Hence, researchers who are interested in using the social scales at this young age should consider the relevance of these questions in their daily experience.

Furthermore, as expected, more subscales demonstrated acceptable internal consistencies at T2 than T1, while some subscales (e.g., depression, overanxious, ODP, and mother and father hostility) had poor internal consistencies at both timepoints (Cronbach’s alpha < .60). The low internal consistency of depression and parenting has been reported in previous studies, especially among younger children (Albow et al., 1999; Coldwell et al., 2006). The internal consistency of the parenting subscales at T2 in the current study may be lower than expected due to the smaller sample size and removal of some items in the online data collection during the COVID-19 pandemic. To our knowledge, only one study has reported the Cronbach’s alpha of overanxious (0.62) and none has reported that of ODP subscale. Hence, more studies are needed to corroborate the finding of poor reliabilities for these subscales.

#### Cross-informant Agreement

Child-report BPI symptomatology broad and subscales both demonstrated some levels of agreements with caregivers’ reports across times. Specifically, internalizing symptoms broad scales showed associations with AC’s reports at T1 and maternal reports at T2, whereas the externalizing broad scale was only associated with ACs’ reports at T2. Among all the BPI symptomatology subscales, separation anxiety showed the strongest cross-informant agreement, linking with both mothers’ and ACs’ reports at both timepoints. Although ample past studies have already demonstrated of the agreement between child-report via BPI and maternal report, our current study provided additional evidence by including AC reports, which also indicated good agreement with children’s reports through the BPI. Given that anxiety and ODP are among the most prevalent psychiatric disorder in early childhood (De Los Reyes et al., 2015; Dougherty et al., 2013), it is encouraging to add evidence to the concordance between child-report via BPI and parental report for assessing symptoms related to these disorders at an early age. At the age of 5, children’s reports of their own depression and social inhibition were significantly related to reports from both parents. Interestingly, depressive symptoms reported by children at age 5 were significantly related to parental reports of anger and aggression, which was in line with previous findings that depression was associated with early onset externalizing behaviors in young children (Loth et al., 2014). This highlights the importance of obtaining children’s perception of their own depressive symptoms, which could be manifested as behaviors that are perceived as externalizing problems from adult’s perspective.

Child-report on the BPI parenting broad scale showed cross-informant agreement at both timepoints. Interestingly, child reports of parenting behaviors were only significantly associated with reports of ACs but not of mothers. This is in line with past studies that suggest mothers’ report of own parenting behaviors could be biased (Sessa et al., 2001) and that report of parenting behaviors from ACs such as fathers and grandparents were more aligned with child-perception of parenting. Specifically, it is intriguing that child-report of maternal parenting was significantly correlated with AC’s reports of their own parenting behaviors. This could be reflecting a similarity in parenting behaviors across both parents, or at least that in the child’s perceptions of behaviors from both parents. The former is supported by the positive correlations between maternal and AC’s reports of parenting (*r* = .22 – .47; see Supplementary Figure S6 for full correlation heatmap), and the latter is supported by the high correlations between BPI maternal and paternal emotional warmth as well as hostility (*r* = 0.54 and 0.56, respectively; Figure S7). These findings support the value of obtaining multi-informant reports that provide converging but different information that may be differentially linked to child-reports.

Stronger cross-informant agreement was found at age 5 than age 4 in numerous scales in the current study, as indicated by the significant associations between all four broad scales with similar constructs reported by either one or both parents at age 5. This suggested that as children grew older, their perception of their own symptoms and behaviors were more aligned with their parents’ perspective. This is consistent with past findings that showed good construct validity of the BPI broad scales of internalizing and externalizing problems with older children (Luby et al., 2007; Ringoot et al., 2013, 2017) and extended the previous literature to show that the social broad scale also showed good construct validity with children at age of 5.

To further our understanding of the changes or similarities in BPI outcomes across a one-year gap, we capitalized on our longitudinal design to assess the stability of the BPI scales over time. We found that social and parenting subscales showed significant correlations over time, which was consistent with past studies that examined stability over time or test-retest reliability of BPI broad scales (Arseneault et al., 2005; Stone et al., 2014). Extending past literature, we also found that child-report of prosocial behavior and father emotional warmth showed stability over time. In line with the literature on trajectory of children’s symptomatology in early age (Basten et al., 2016), our findings indicated some instability of children’s internalizing and externalizing symptoms. Notably, the COVID-19 pandemic occurred between T1 and T2, which has been documented to affect children’s psychological adjustment (Foley et al., 2022). Hence, these findings have to be corroborated by future studies outside of the context of the pandemic.

#### Recommendations for Using BPI with Young Preschoolers

We offered a number of recommendations to guide future researchers who are interested in using BPI with children at age 4. First, given our findings that children at age 4 seemed to show lower level of concentration during BPI, researchers should consider reducing the number of items asked in the interview or allow more breaks to ensure children maintain a good level of concentration throughout the whole interview (Ablow & Measelle, 2018). Second, our results suggested that broad scales had better reliability (internal consistency and stability over time). Hence, it may be more appropriate to use broad scales for analysis if possible. However, if researchers are more interested in using individual subscales for specific research questions, we found that overt hostility appeared to show acceptable internal consistency at age 4, while separation anxiety showed good cross-informant agreement and stability. Lastly, social scales, including peer acceptance/rejection and social inhibition, may not be suitable for assessing social behaviors at age 4 as it showed poor reliability and cross-informant agreement.

#### Limitations

The current study extended the literature by comparing the feasibility, reliability, and cross-informant agreement of self-reporting of children at age 4 and 5 using BPI. Our study had several strengths including the use of multi-informant data and longitudinal design. However, several limitations should be discussed. First, our sample was predominantly economically advantaged and highly educated White American families, which limited the generalizability to families with different socioeconomic and racial backgrounds. Also, our sample size was too small for advanced analyses such as factor analysis (MacCallum et al., 1999), therefore we were not able to confirm the factor structure of the scales. Nonetheless, numerous previous studies have established the factor structure of the scales we used in the current study (Agarwal et al., 2016; Ringoot et al., 2013). Future studies with larger sample size using multi-informant data may also consider testing measurement invariance across informants to ensure that any discrepancies between informants are due to actual differences rather than measurement errors. Furthermore, although our samples included both depressed and nondepressed mothers, we did not focus on the comparison of the BPI results between the two groups given the focus of the current paper is to evaluate the methodological strengths and weaknesses of using the BPI at different ages. An interesting future direction will be to investigate how child-report may differ between those with depressed and nondepressed mothers.

Second, due to the global pandemic, our data collection for T2 was delayed and some participants completed the T2 visit online, including BPI (*n* = 11). It is unclear how the psychometric properties of BPI may be impacted due to the changes in data collection method. Nevertheless, when looking at responses from the 11 children who completed BPI online, both the number of items that are skipped (max. = 1 items) and not codable responses (max. = 2 items) were low. Third, different methods were used for child reports (interview) and parental reports (questionnaire). Additionally, the items used in child and parental reports were not the same even though they may assess similar underlying constructs, especially for the symptomatology and parenting scales. There was no parental report that directly measured the same constructs of children’s social behaviors as the BPI social scales, especially peer acceptance/rejection and prosocial behaviors. Hence, the lack of significant correlations between child and parental reports for some of the BPI scales should be interpreted with caution. Future research examining the construct validity of the social scales may include parental reports that assess the same construct. Furthermore, the global impression codes captured subjective impression of the coders; although an acceptable level of inter-coder reliability for these codes was found in the current study, it is important to note that this scale was originally designed to monitor data quality and each dimension was measured with only one item. Without further details of its psychometric properties and past data for references, caution should be used when interpreting these results.

## Conclusion

The current study was one of the first few studies that investigated the changes in BPI performance across children of age 4 and 5 based on three criteria: feasibility, reliability, and cross-informant agreement. Although unsurprisingly children provided more reliable responses and showed significantly higher levels of concentration and believability at age 5, most broad scales and certain subscales showed good reliability (e.g., Overt Hostility/aggression) and good cross-informant agreement (e.g., separation anxiety) at age 4. We also found that child-report was more often associated with report from ACs than maternal report, which highlighted the importance of including perspectives from other caregivers when studying socioemotional development and parenting in young children. More empirical studies are needed to corroborate our findings and continue evaluating the possibility of using BPI with young children. Nevertheless, our study supported the exciting and promising possibility of using puppets to assess young children’s perceptions of own experience and behaviors, potentially supporting earlier detection of symptoms or more child-centered understanding of social experiences to inform appropriate interventions that may help prevent more severe problems in the future.

## Figures and Tables

**Table 1:** Global impression scores on children’s performance during BPI

	T1 (N = 123)			T2 (N = 89)			T2-T1 (n = 89)	
	*Mean*	*SD*	Range	*Mean*	*SD*	Range	*t*	*P*
Believability	4.96	1.10	2–7	5.70	0.84	2–7	**4.72**	0.000
Concentration	4.58	1.73	1–7	5.25	1.49	1–7	**3.26**	0.002
Understanding	5.25	0.97	2–6	5.63	1.01	3–7	**2.67**	0.009
Consistency	4.55	1.08	1–7	5.49	0.76	3–7	**7.48**	0.000
Distress (social)	5.16	1.03	2–7	5.28	1.13	3–7	0.87	0.390
Distress (parenting)	5.21	1.02	2–7	5.31	1.00	3–7	0.67	0.502

*Note*. Higher score indicated better performance and fewer problems

**Table 2:** Mean Scores, Cronbach’s alphas, and correlations of scales and subscales at T1 and T2

	T1 (N = 123)		T2 (N = 89)		Stability	
Scale	*Mean (SD)*	a	*Mean (SD)*	a	*r*	*p*
A. Internalizing	4.77 (0.83)	0.78	4.70 (0.82)	0.78	0.19	0.08
B. Externalizing	5.00 (0.83)	0.74	5.42 (0.70)	0.72	0.10	0.35
C. Social	4.58 (0.75)	0.54	4.70 (0.82)	0.78	**0.34**	0.00
D. Parenting	5.18 (0.61)	0.74	5.31 (0.55)	0.73	**0.35**	0.00
1. Depression	4.91 (0.89)	0.46	5.14 (0.83)	0.51	0.21	0.06
2. Separation Anxiety	4.54 (1.30)	0.57	4.18 (1.29)	0.64	**0.26**	0.02
3. Overanxious	4.84 (104)	0.57	4.79 (0.92)	0.59	0.07	0.53
4. Oppositional Defiant	4.85 (1.01)	0.35	5.29 (0.83)	0.60	0.06	0.62
5. Overt Hostility/aggression	5.16 (1.01)	0.72	5.51 (0.81)	0.60	−0.01	0.93
6. Prosocial Behaviors	4.08 (1.24)	0.67	4.90 (1.13)	0.75	**0.39**	0.00
7. Peer acceptance/rejection	5.14 (0.94)	0.26	5.25 (0.95)	0.61	0.03	0.83
8. Social inhibition	4.58 (1.09)	0.50	4.06 (1.20)	0.69	0.09	0.41
9. Mother Emotional Warmth	5.39 (0.84)	0.41	5.58 (0.82)	0.71	0.19	0.08
10. Father Emotional Warmth	5.07 (117)	0.63	5.46 (0.94)	0.68	**0.39**	0.00
11. Mother Hostility	5.16 (0.91)	0.60	5.11 (0.83)	0.52	**0.23**	0.04
12. Father Hostility	5.06 (0.95)	0.51	5.05 (0.96)	0.54	0.08	0.46

*Note*. A higher score indicates more positive perception and fewer problems. Stability = correlation between T1 and T2.; a = Cronbach’s a. *p < .05
